# Extra-axial cranial nerve enhancement: a pattern-based approach

**DOI:** 10.1007/s11547-023-01734-2

**Published:** 2023-10-26

**Authors:** Giulia Moltoni, Andrea Romano, Antonella Blandino, Serena Palizzi, Allegra Romano, Benedetta D’Arrigo, Alessia Guarnera, Francesco Dellepiane, Valentina Frezza, Olga Gagliardo, Francesca Tari Capone, Andrea Grossi, Guido Trasimeni, Alessandro Bozzao

**Affiliations:** 1 NESMOS, Department of Neuroradiology, S.Andrea Hospital, University Sapienza, Via di Grottarossa, 00135 Rome, Italy; 2 Imaging Department, San Paolo Hospital, Civitavecchia, Italy; 3 Neuroradiology Unit, Imaging Department, Bambino Gesù Children’s Hospital, IRCCS, Piazza di Sant’Onofrio 4, 00165 Rome, Italy

**Keywords:** Cranial nerve enhancement, MRI, Tumoral spread, Infective diseases, Inflammatory diseases

## Abstract

Cranial nerve enhancement is a common and challenging MRI finding that requires a meticulous and systematic evaluation to identify the correct diagnosis. Literature mainly describes the various pathologies with the associated clinic-radiological characteristics, while the radiologist often needs a reverse approach that starts from the radiological findings to reach the diagnosis. Therefore, our aim is to provide a new and practical pattern-based approach to cranial nerve enhancement, which starts from the radiological findings and follows pattern-driven pipelines to navigate through multiple differential diagnoses, guiding the radiologist to reach the proper diagnosis. Firstly, we reviewed the literature and identified four patterns to categorize the main pathologies presenting with cranial nerve enhancement: unilateral linear pattern, bilateral linear pattern, unilateral thickened pattern, and bilateral thickened pattern. For each pattern, we describe the underlying pathogenic origin, and the main radiological features are displayed through high-quality MRI images and illustrative panels. A suggested MRI protocol for studying cranial nerve enhancement is also provided. In conclusion, our approach for cranial nerve enhancement aims to be an easy tool immediately applicable to clinical practice for converting challenging findings into specific pathological patterns.

## Background

Cranial nerves (CNs) are twelve paired sets of nerves with sensory and/or motor functions. The first (olfactory nerve—CN I) and second pair (optic nerve—CN II) are considered extensions of the central nervous system arising from the telencephalon and diencephalon, respectively; the other ten pairs of CN originate from the brainstem having their nuclei in the midbrain (oculomotor nerve—CN III and trochlear nerve—CN IV); pons (trigeminal nerve—CN V, abducens nerve—CN VI, facial nerve—CN VII, vestibulocochlear nerve—CN VIII), and medulla oblongata (glossopharyngeal nerve—CN IX, vagus nerve—CN X, accessory nerve—CN XI, hypoglossal nerve—CN XII). From their origin, CN is usually divided into cisternal, intracranial, and extra-cranial segments, leaving the central nervous system through cranial foraminal. Microscopically, CN is surrounded by connective tissue sheaths including endoneurium, perineurium, and epineurium. Axons of each nerve, except I and II, are myelinated by Schwann cells. Tight junctions present in the endothelium of the endoneurial capillaries and inner layers of the perineurium guarantee the integrity of the blood-nerve barrier (BNB). The loss of BNB structural integrity caused by pathogenic events involves leakage and accumulation of contrast material, which results in pathological enhancement alone or associated thickening if tumoral or inflammatory infiltrates occur [1]. Cranial nerve enhancement (CNE) often represents a diagnostic challenge, being not always easy to distinguish among several causes, such as neoplasms, inflammation, autoimmune diseases, demyelination, infections, traumas, ischemia or radiation.

The pitfalls of evaluating CNE on MRI may be related to the slenderness of the nerves and use of suboptimal imaging parameters, which could lead to under-detection, while the presence of perineural vascular plexuses could lead to overestimation. Indeed, although cranial nerves physiologically have no contrast enhancement, some segments do have perineural vascular plexuses that may cause apparent moderate enhancement derived by this kind of vascular organization. Typically, the trigeminal ganglion and the proximal portions of its divisions V2 and V3, the geniculate, tympanic, and mastoid segments of the facial nerve, and the intracanal segment of the hypoglossal nerve are surrounded by enhancing perineural vascular plexuses [2–4].

Our purpose is to provide a useful tool to facilitate differential diagnosis of pathologies causing extra-axial CNE through a pattern-based approach.

We tried to organize the most common disorders that can affect cranial nerves with consequent pathological enhancement by mapping them out in a flowchart by cross-referencing data with the literature.

With the help of the flowchart we developed, it is possible to narrow the range of possible diagnoses based on the identification of the affected cranial nerves and their pattern of enhancement (mono or bilateral, linear or thickened).

Additionally, to correlate the type of enhancement to the pathogenetic mechanisms, we linked the pattern of CNE and the corresponding cranial nerve involvement with each of the most prevalent pathologies potentially causing CNE, grouped into three macro-sets: infections, inflammatory diseases, and tumor-related enhancement. Primary CN tumors (i.e., neurinoma) will not be included because they are space-occupying lesions with a completely different appearance from the other types of CNE here discussed.

## Data collection

This is a narrative review, and we revised the literature to evaluate the presence of CNE, its pathologic causes, and its appearance. Two independent and experienced neuroradiologists (A.R. 15 years of experience and G.M. 5 years of experience) reviewed all English-language original articles and case reports inherent to the topic, excluding articles about CN space-occupying lesions.

### Imaging of cranial nerve enhancement

Among imaging techniques, magnetic resonance (MR) has been referred to as the gold standard for the evaluation of cranial nerve pathology. Usually, the sequence of choice to depict the pathological enhancement of a cranial nerve after gadolinium-based contrast agents administration is a fat-suppressed high-resolution three-dimensional T1-weighted fast gradient echo (3D T1 FGRE). This high-resolution volumetric sequence would be preferable to in-plane T1 spin-echo weighted images, allowing multiplanar reconstruction and evaluation of thin structures such as cranial nerves [5]. Nevertheless, this sequence may present some limitations, such as the presence of the near venous plexuses that may mask a pathological enhancement [1].

Due to complete cerebrospinal fluid suppression and to prevent flow artifacts around the brainstem, the use of fat-saturated three-dimensional fluid-attenuated inversion recovery sequence (3D FLAIR) after contrast medium injection has been studied. It performs better than contrast-enhanced T1-weighted images in the detection of cranial nerves and roots attached to the brainstem [6–8].

Recently, a contrast-enhanced 3D-T1-turbo spin-echo (TSE) black-blood sequence has gained attention, showing optimal diagnostic performance in depicting cranial nerve enhancement as it suppresses signals from vessels, including the near venous plexuses, and provides an increased contrast-to-noise ratio [9, 10].

Beyond the contrast enhancement, it should be kept in mind that studying a pathology involving cranial nerves (CNs) also means properly evaluating cranial nerves anatomy and morphology by a pre-contrast high-resolution three-dimensional heavily T2-weighted sequence (such as SPACE/CISS/FIESTA-C/VISTA/Cube) and the eventual pre-contrast abnormal cranial nerve signal usually by a pre-contrast fat-saturated 3D FLAIR [11, 12].

Finally, a coronal or axial T2-weighted sequence could be useful to detect denervation changes of facial muscles, representing an indirect sign of cranial nerve pathological involvement, and a pre-contrast T1-weighted spin-echo sequence, usually in the axial plane, may be useful for the evaluation of fat invasion in pathologies involving the extraforaminal segments of cranial nerves [5, 11].

Due to its low-contrast resolution, computed tomography (CT) is inferior to MRI for the visualization of cranial nerve pathology; however, it can be used in addition to MRI for the assessment of the morphology of the foramina and intraosseous pathways of cranial nerves at the skull base [5].

In Table 1 (Table 1), we proposed an MRI protocol for studying CNE enhancement.

**Table 1:** Proposed MRI protocol for the evaluation of cranial nerve pathology (1–12)

MRI sequences	Indication
*Pre-contrast*	
3D FLAIR	Lesion characterization
3D high-resolution heavily T2WI*	Anatomical definition
Coronal/Axial T2 WI	Denervation changes
Axial T1 WI	Fat invasion (extraforaminal CN segments)
*Post-contrast*	
3D T1 fast gradient-echo**	Cranial nerve enhancement
or	
3D T1-turbo spin-echo black-blood	Cranial nerve enhancement, advantage: suppression of vessels signals
or	
3D FLAIR (optional)	Cranial nerve enhancement, advantages: no flow artifacts and better evaluation of leptomeningeal involvement

WI, weighted image; CN, cranial nerve; FLAIR, fluid-attenuated inversion recovery; SPACE, sampling perfection with application optimized contrast using different flip angle evolution; CISS, constructive interference in a steady state; FIESTA, fast imaging employing steady-state acquisition; VISTA, volume isotropic turbo spin-echo acquisition; MPRAGE, Magnetization Prepared-RApid Gradient Echo; VIBE, volumetric interpolated breath-hold examination; FSPGR, fast spoiled gradient echo; THRIVE, T1-weighted high-resolution isotropic volume examination

*SPACE (Siemens), CISS/FIESTA-C/Cube (GE), VISTA (Philips)

**MPRAGE/VIBE (Siemens), FSPGR (GE), THRIVE (Philips)

### Cranial nerve enhancement patterns

**Fig. 1:**
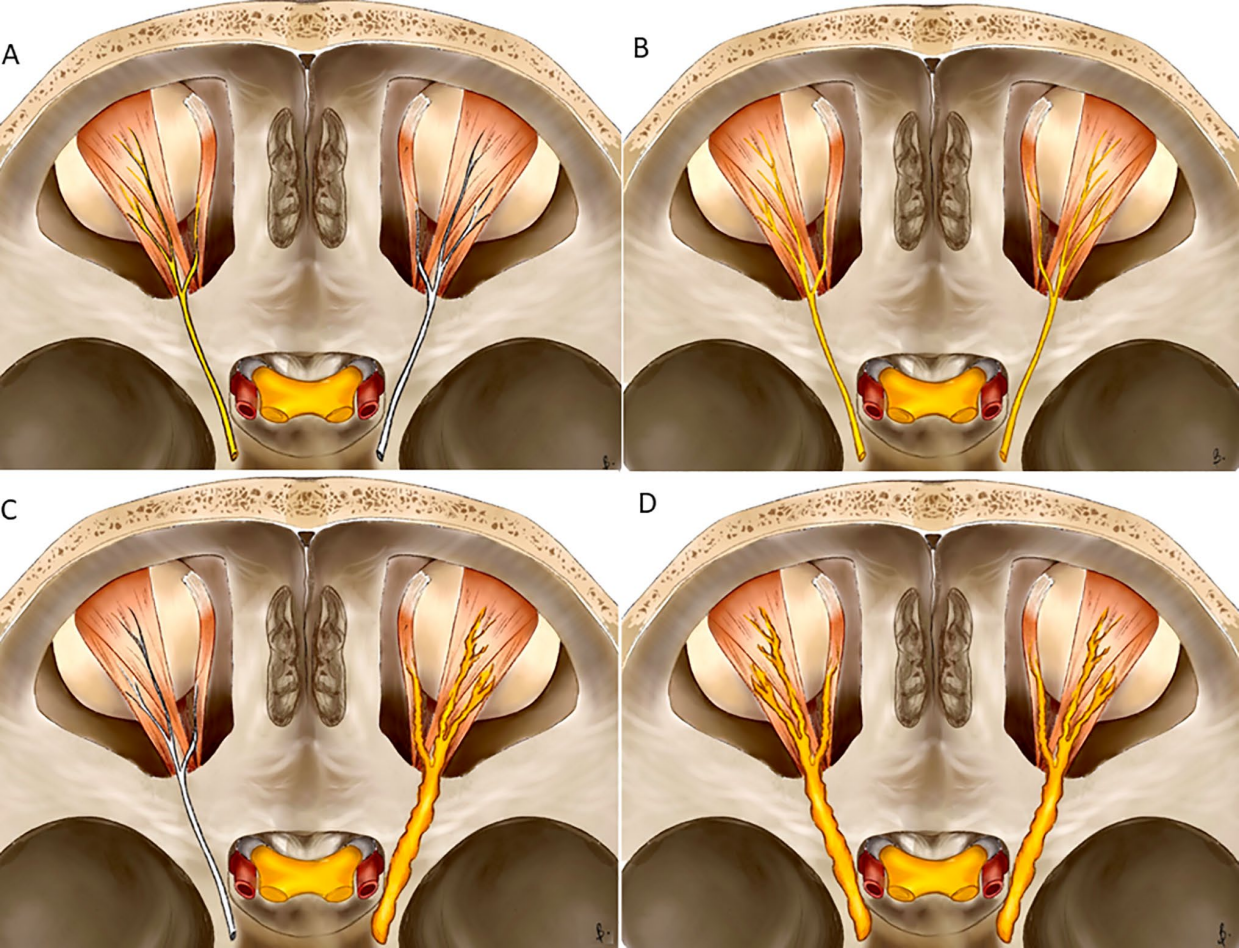
Cranial nerve enhancement patterns: **a** unilateral linear, **b** bilateral linear, **c** unilateral thickened, **d** bilateral thickened. Where, by linear CNE, we mean a post-contrast enhancement along the nerve, or a part of it, without an increase in the diameter of the nerve, and by thickened enhancement, we mean a post-contrast enhancement along the nerve, or a part of it, with an associated increased nerve diameter that is often irregular or nodular

We identified two types of CNE: linear and thickened. Where, by linear CNE, we mean a post-contrast enhancement along the nerve, or a part of it, without an increase in the diameter of the nerve, and by thickened enhancement, we mean a post-contrast enhancement along the nerve, or a part of it, with an associated increased nerve diameter that is often irregular or nodular. Each of these types of enhancement can involve one or more cranial nerves, even bilaterally; therefore, we identified the following four patterns of CNE (Fig. 1):

1. unilateral linear CNE
2. bilateral linear CNE
3. unilateral thickened CNE
4. bilateral thickened CNE

**Fig. 2:**
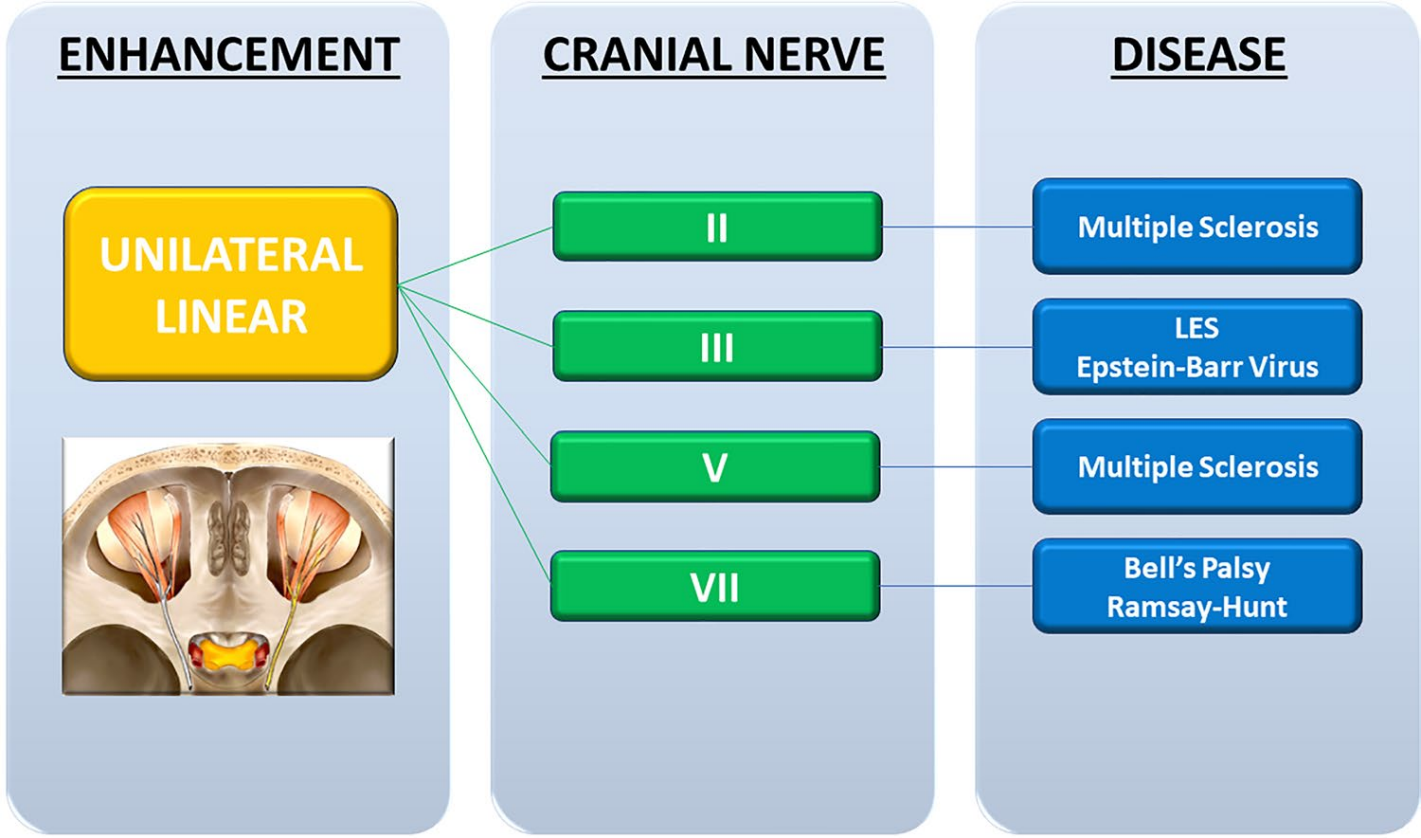
Unilateral linear pattern flowchart with the type of cranial nerve involved and the possibly related pathological condition

**Fig. 3:**
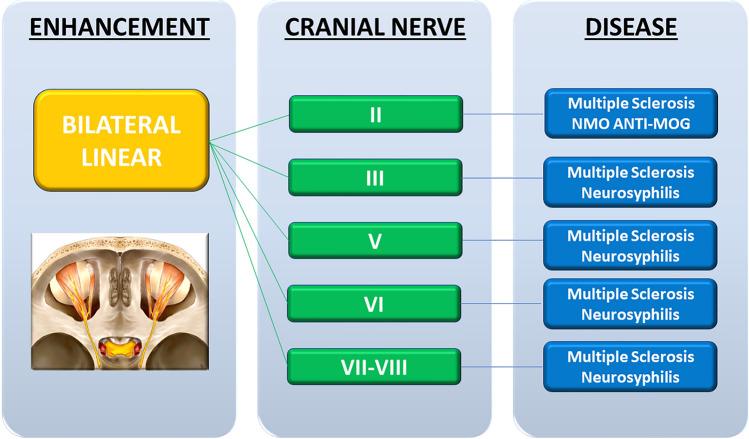
Bilateral linear pattern flowchart with the type of cranial nerve involved and the possibly related pathological condition

**Fig. 4:**
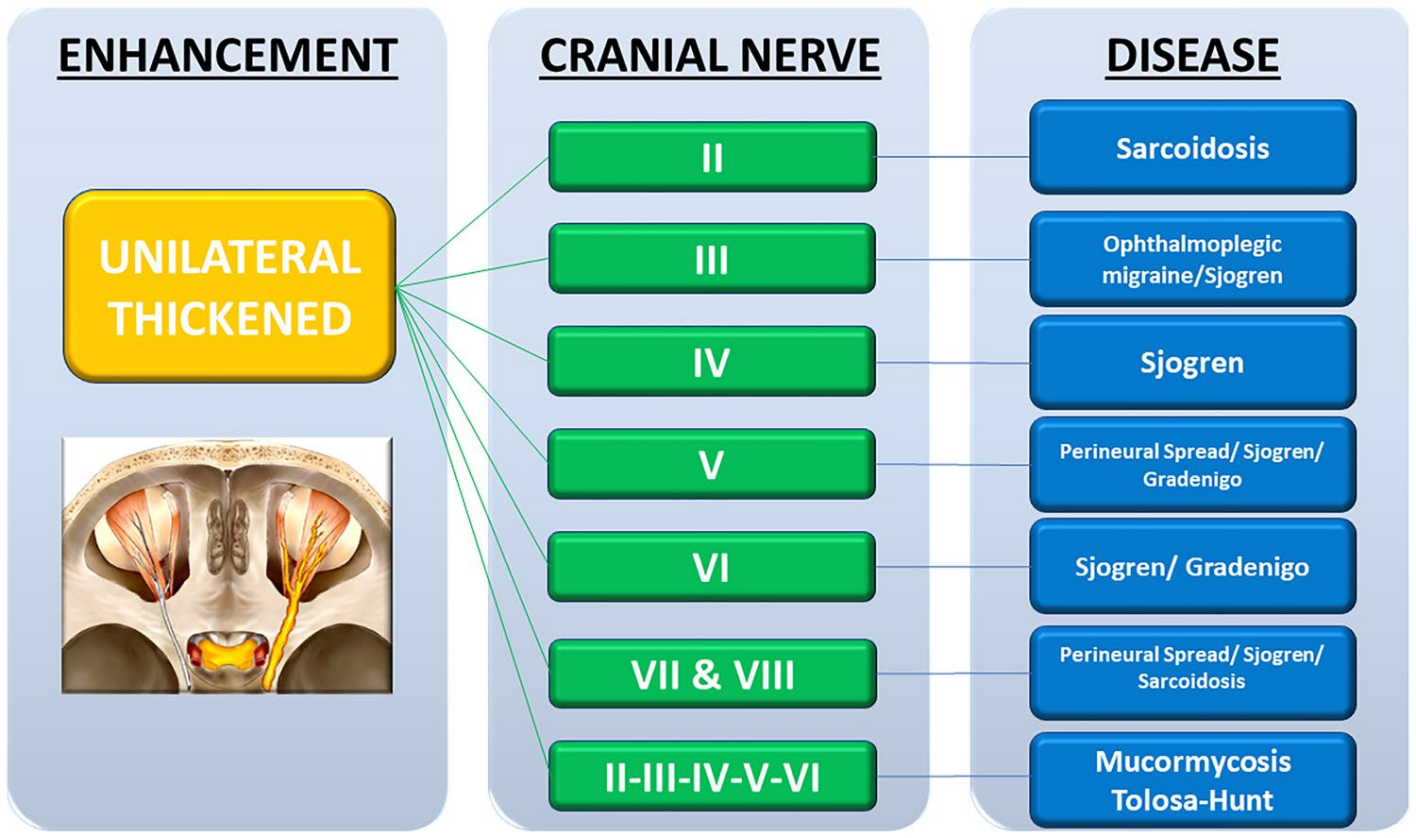
Unilateral thickened pattern flowchart with the type of cranial nerve involved and the possibly related pathological condition

**Fig. 5:**
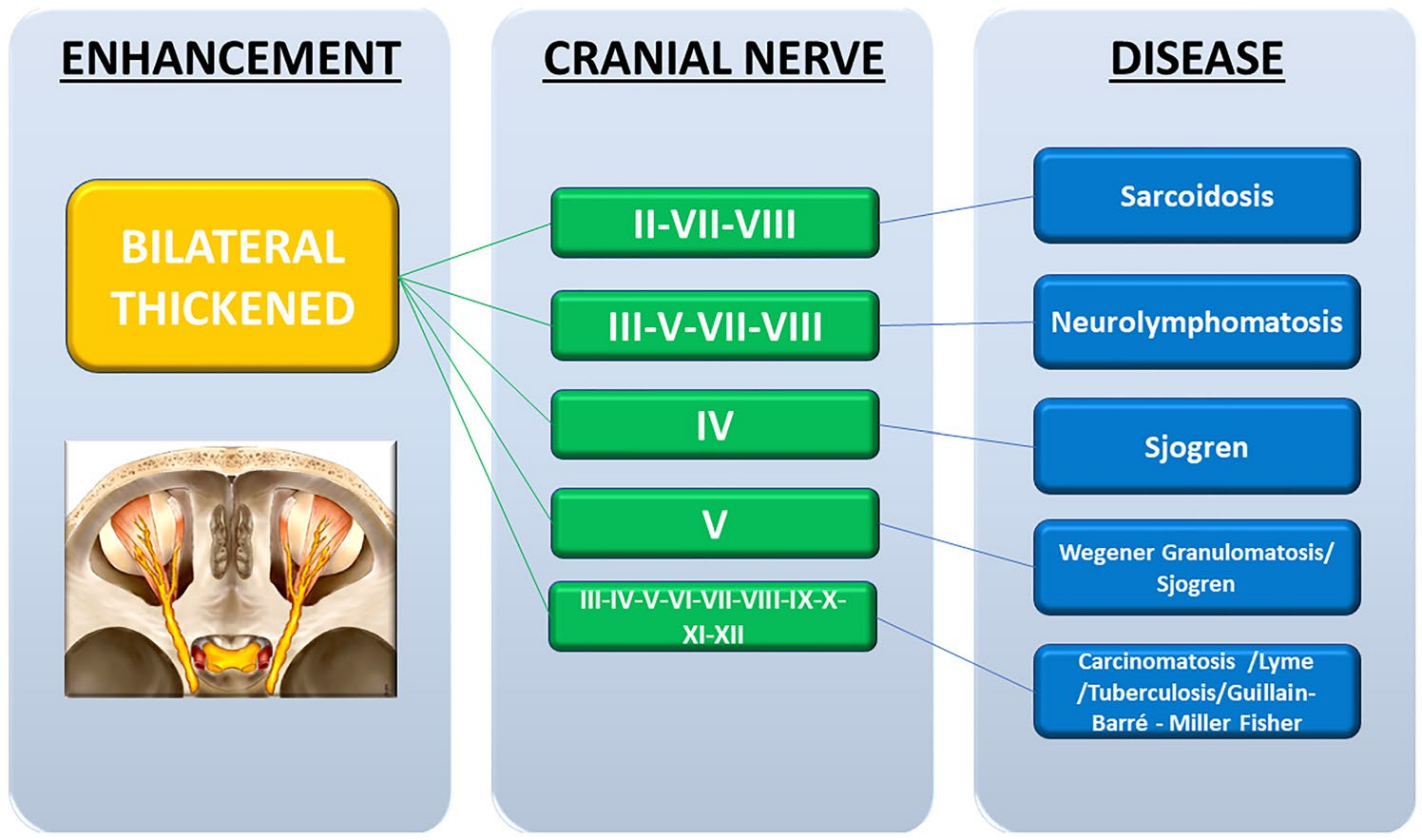
Bilateral thickened pattern flowchart with the type of cranial nerve involved and the possibly related pathological condition

In the flowcharts (Figs. 2, 3, 4, 5), we reported each kind of CNE pattern with the type of cranial nerve involved and the possibly related pathological condition.

The two linear enhancement patterns are related to a select number of illnesses, most of which are brought on by inflammatory or infectious conditions; only multiple sclerosis, among these illnesses, may appear with both bilateral and unilateral linear patterns and may affect a significant number of cranial nerves [13]. A linear CNE pattern is unrelated to any oncologic disorders.

The thickened CNE patterns offer a more challenging condition because a range of oncologic, inflammatory, and infectious diseases can justify this type of enhancement. Bilateral thickened enhancement is mainly related to neoplastic carcinomatosis [14], infective conditions such as tuberculosis or Lyme disease [15, 16] and inflammatory/immuno-mediated pathologies such as Guillain–Barré syndrome and its variant Miller–Fischer syndrome [17, 18].

Some inflammatory/immuno-mediated conditions such as Sarcoidosis and Sjogren's disease are more pleomorphic, with both unilateral and bilateral cranial nerve involvement [19, 20]. Unilateral thickened pattern could be the expression of a perineural spread, especially if the V cranial nerve and the VII–XII nerves are involved [21].

An ophthalmoplegic migraine or the Tolosa–Hunt disease may be suspected if oculomotor nerves are affected [22, 23].

Although we only listed the most common pathologies and underlined that the final diagnosis can only be reached with the whole set of clinical and laboratory findings and the whole set of MRI, the proposed flow chart aims to represent a practical approach in guiding the radiologist toward a probable etiological cause of cranial nerve enhancement.

## Pathogenesis: why can cranial nerve enhancement occur?

### Tumor-related enhancement

Malignant tumors can cause CNE mainly through two routes of tumor spread: perineural tumor spread (PNTS) and leptomeningeal carcinomatosis (LC)/neurolymphomatosis. The MRI detection of these pathological conditions is crucial because they correlate with decreased survival, and PNTS also increases locoregional recurrence.

Malignant cells can dissociate from the primary tumor and establish metastatic deposits at nearby or distant sites. Metastatic deposits in and along nerves result in pathological enhancement and thickening, commonly with a micronodular appearance. Due to the pathogenic mechanisms, cranial nerve involvement is generally unilateral in the case of perineural spread and bilateral in leptomeningeal carcinomatosis and neurolymphomatosis [24, 34].

A malignancy spreads centripetally from the initial tumor site into the central nervous system using the support of a nearby nerve in a process known as perineural tumor spread (PNTS). The term perineural tumor invasion (PNTI) is often mistakenly used as a synonym for PNTS. It is important to clarify that while PNTI refers to a histologic finding of tumor cell infiltration, PNTS refers to the macroscopic involvement, radiologically apparent with a sensitivity of 95% on MRI [25].

In the literature, it is reported commonly in head and neck cancer, reaching up to 50–70% in patients affected by mucosal squamous cell cancer and in at least half of patients affected by adenoid cystic carcinoma [21, 25].

The exact mechanism by which PNTS occurs is unclear, but modern studies have demonstrated that it is the result of a dynamic molecular process involving active crosstalk between the tumor and nerve cells [25, 26]. It has been proven that proteins involved in neural homeostasis, axonogenesis, and dendritic growth play a key role in cancer cell proliferation, perineural invasion, and migration. These proteins are numerous including brain-derived neurotrophic factor, nerve growth factor, neurotrophin-3, neurotrophin-4, glial cell line-derived neurotrophic factor, the neural cell adhesion molecule, substance P, laminin-5, semaphorins, and their receptors [26, 27].

**Fig. 6:**
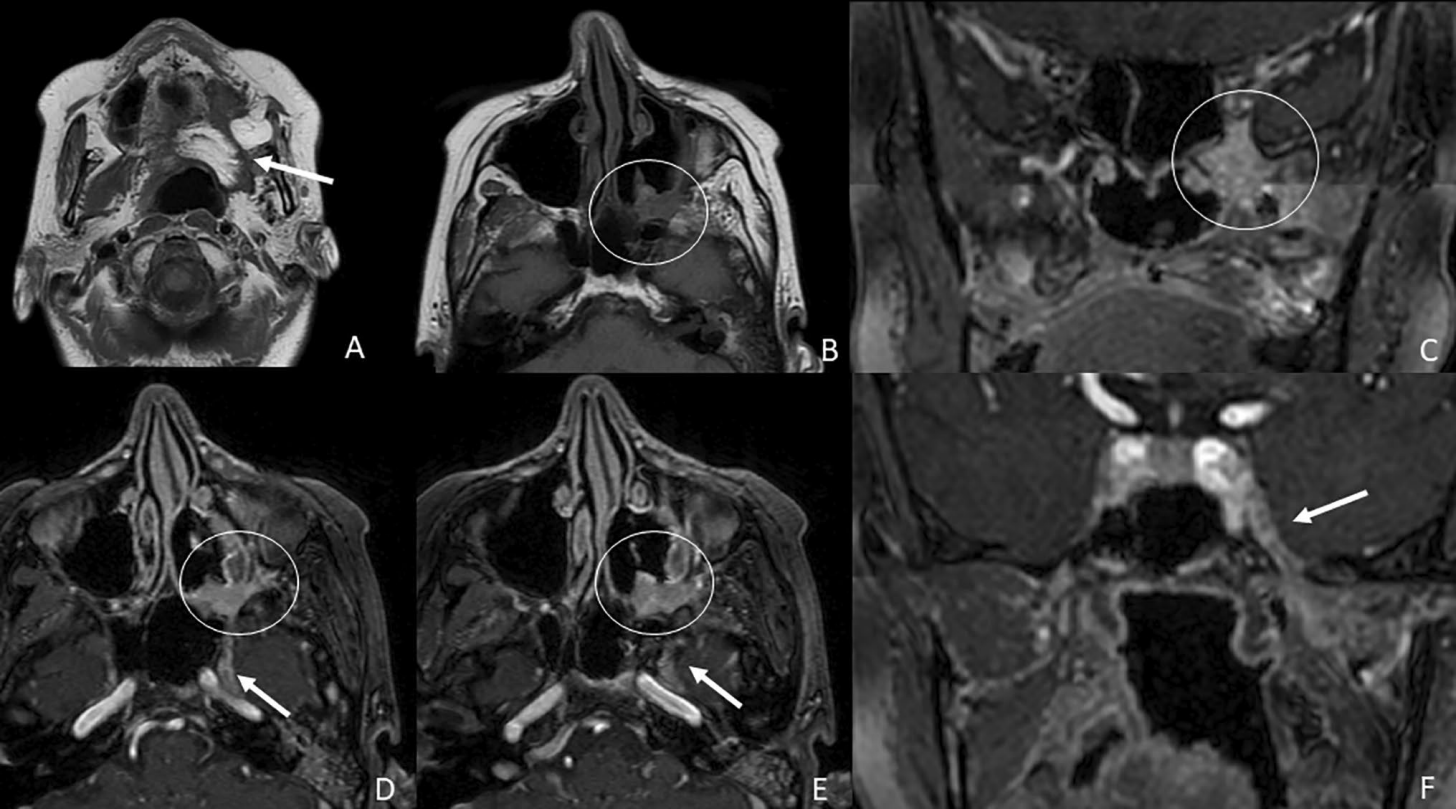
Patient with a history of oral cavity cancer treated by left hemiglossectomy with temporal flap reconstruction visible in **a**. (arrow) axial T1-weighted image. The axial T1-weighted image in **b** shows obliteration of the left pterygopalatine fossa by a pathological enhancing tissue well visible in **c** coronal fat-saturated 3D T1 post-contrastographic weighted images (circle). In **d** axial fat-saturated 3D T1 post-contrastographic weighted images it is visible a thickened enhancement extending to the V2 segment of the left trigeminal nerve (arrow) and in **e** axial and **f** coronal fat-saturated 3D T1 post-contrastographic weighted images a thickened enhancement of the ipsilateral V3 segment (arrow). These findings are consistent with perineural spread

Since it is typically a unilateral process, the most characteristic MRI finding of PNTS is an asymmetrical enlargement and enhancement (monolateral thickened pattern) of the involved cranial nerve compared to the healthy contralateral nerve. Additional findings include obliteration of perineural fat planes, denervation changes, and homolateral widening of foramina [25] (Fig. 6).

Although skip lesions are uncommon, they are theoretically possible in the case of PNTS, where the tumor tissue is often continuous down the nerve [25, 27].

Due to its size, cranial nerve V is the most affected nerve. Yet, cranial nerves III, IV, and VI can all be affected because of their intracavernous tracts' location within CN V [25].

Another possible cause of CNE in patients with head and neck tumors, in differential diagnosis with PNTS, is related to complications of radiotherapy causing the so-called radiation-induced neuritis. Radiotherapy may induce loss of BNB integrity due to demyelination, ischemia, coagulation necrosis, or peripheral fibrosis. In radiation-induced neuritis, the affected nerves usually appear thickened with T2 hyperintense signal and enhancement. Although all cranial nerves could be potentially involved, depending on the radiation exposure, literature data reported the XII cranial nerve as the most affected nerve following irradiation for nasopharyngeal carcinoma. Two conditions could help in differentiating radiation-induced neuritis from PNTS, the type of CNE usually more nodular in PNTS, and the timing of onset usually months to years after radiation therapy exposure in radiation-induced neuritis [1].

**Fig. 7:**
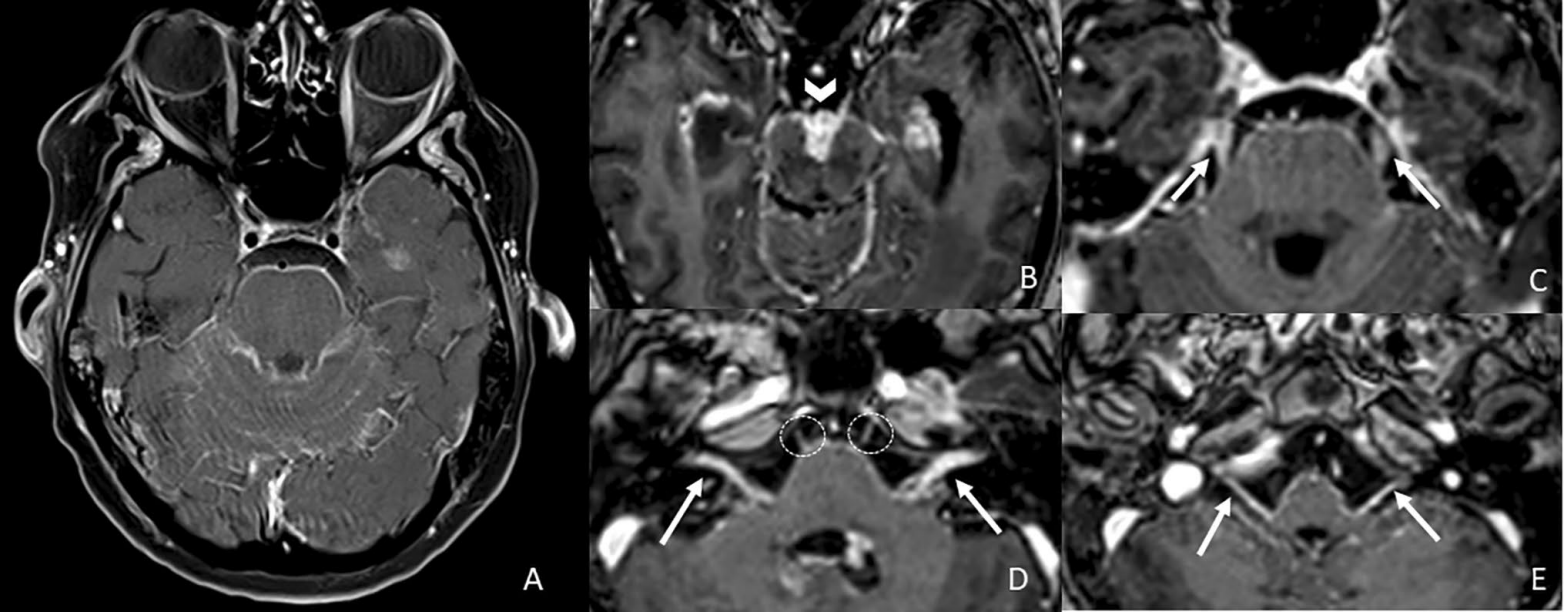
Axial post-contrastographic T1 3D-weighted images showing diffuse leptomeningeal carcinomatosis in a patient affected by melanoma (**a**), with bilateral thickened enhancement of the intracisternal segment of the III (**b**. arrowhead), VI (**c**. arrows), slight of the VI (**d**. circles), VII–VIII (**d**. arrows) and mixed nerves (**e**. arrows)

The other route of tumor spread already mentioned is leptomeningeal carcinomatosis. This condition refers to a metastatic involvement of the cerebrospinal fluid and leptomeninges by a central nervous system tumor, any solid systemic tumor, or hematologic malignancy. In the literature, it is reported to occur in 4–15% of patients with malignancy, and, in decreasing order, breast cancer, lung cancer, and melanoma are the most common systemic cancers involved [14] (Fig. 7).

Some theories on how malignant cells reach the leptomeninges include perivascular, arachnoid venous, choroid plexus hematogenous dissemination, contiguous dural metastases, and bone metastases.

The most common MRI sign of leptomeningeal carcinomatosis is pial and subarachnoid space enhancement (linear 32% and micronodular 54%), which is 9% of the time associated with nodular thickening that involves multiple nerves that are distant from each other and thicken bilaterally [28].

Since leptomeningeal carcinomatosis is prone to form larger deposits in areas of CSF stasis, such as the cerebellopontine angle and the peri-mesencephalic cisterns, cranial nerves III, V, VII, and VIII are especially involved [28], but hypothetically all the CN may be affected.

**Fig. 8:**
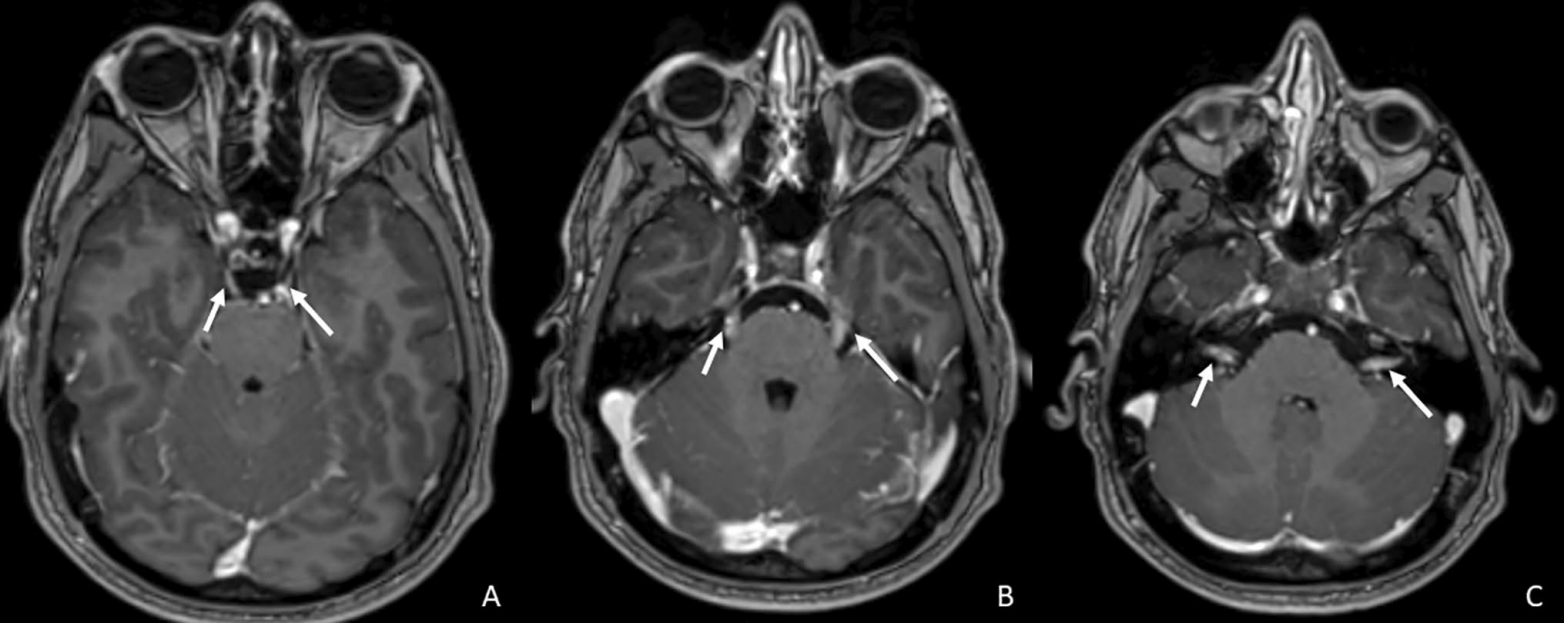
Axial T1 FS post-contrast images showing bilateral thickened enhancement of the intracisternal segment of the III (**a**., arrows), V (**b**., arrows), I and VII–VIII (**c**., arrows) pair of cranial nerves in a patient affected by neurolymphomatosis

Finally, a rare condition that may involve cranial nerves is neurolymphomatosis. It accounts for about the 3% of newly diagnosed non-Hodgkin’s lymphoma or leukemia cases [29]. Different manifestations are described, ranging from a painful polyneuropathy involving the cauda equina to cranial neuropathy and painless or peripheral mononeuropathy involving the sciatic nerve [30]. There is a common involvement of III, V, VI, and VII cranial nerves, with a higher incidence of involvement in their cisternal segments [31]. The enhancement of cranial nerves could be thickened or nodular (bilateral thickening pattern) due to the infiltration of tumor cells into the endoneurium and perineurium [32] (Fig. 8).

### Infective-related enhancement

Cranial nerve enhancement in infectious disorders may result in different patterns; usually, viral infection leads to a unilateral and linear enhancement, whereas bacterial and fungal infections are more likely to give a thickened pattern due to inflammatory or infectious agent cells. In bacterial and fungal diseases, if the cranial nerve is involved by contiguity, the pattern is usually unilateral; if the CNE is the consequence of a disseminated infection, the pattern is bilateral.

Viruses represent the most common infectious disorders affecting cranial nerves and causing viral neuritis [33–35].

**Fig. 9:**
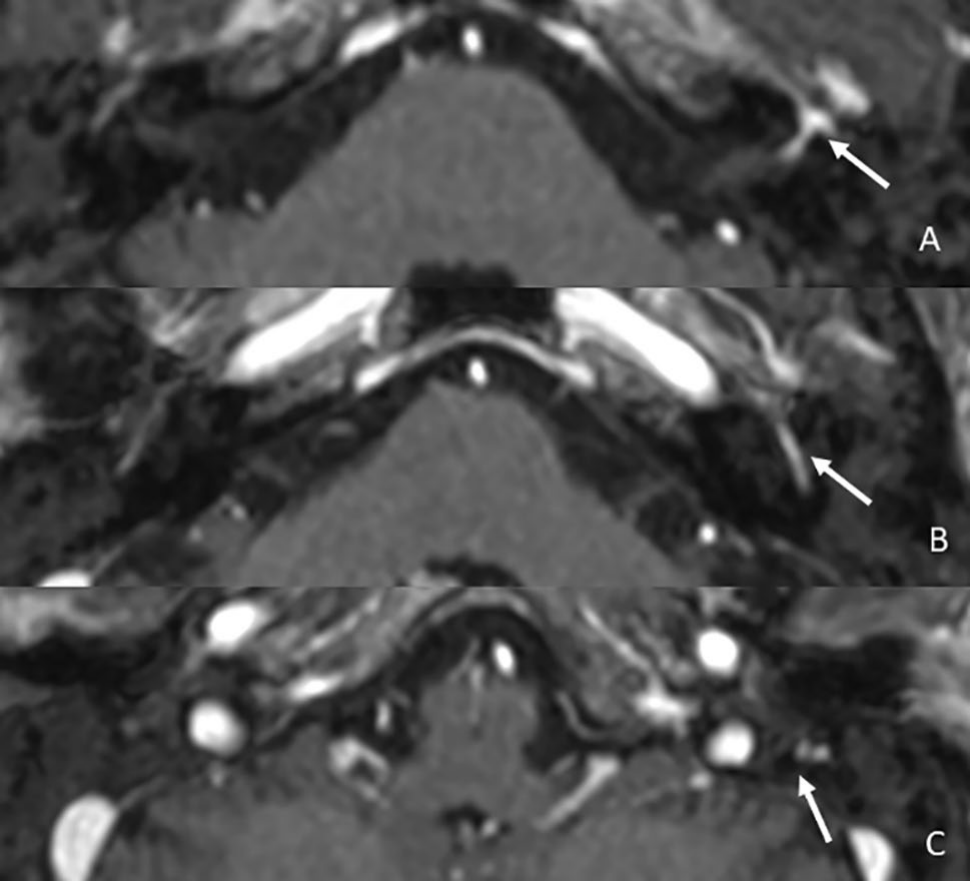
Patient presented with Bell’s Palsy. Axial T1 3D post-contrastographic images showing linear enhancement of the left VII cranial nerve in its geniculate ganglion region (**a**, arrow), intratympanic (**b**, arrow), and intramastoid segments (**c**, arrow)

The most common viruses causing neuritis and consequently CNE belong to the Herpesviridae family, including, among others, Herpes simplex type 1 virus (HSV1), varicella-zoster virus (VZV), and Epstein–Barr virus (EBV). HSV1 and VZV often remain latent in the geniculate ganglion (CN VII) thanks to their neurotropism. If an event causes their reactivation, facial nerve inflammation occurs with blood-nerve barrier breakdown, resulting in a typical monolateral linear CNE [36, 37]; sometimes trans-neural infection of adjacent nerves may be associated [26]. HSV-1 is considered the main etiological cause of Bell’s palsy [38–40] (Fig. 9), while Ramsay Hunt syndrome is caused by VZV reactivation [37]. A slightly different mechanism, probably related to a para-infectious condition rather than a direct viral infection, characterized EBV CN changes that may affect the CN III nerve with the peculiar "shooting star" sign due to the involvement of its root exit zone with adjacent pial enhancement and associated edematous changes in the ventral mesencephalon [41].

Bacterial infection is much less frequent, and CN involvement is often the consequence of other infectious diseases left untreated, generally in the middle ear cavity or paranasal sinuses, or disseminated systemic infection [42–44].

**Fig. 10:**
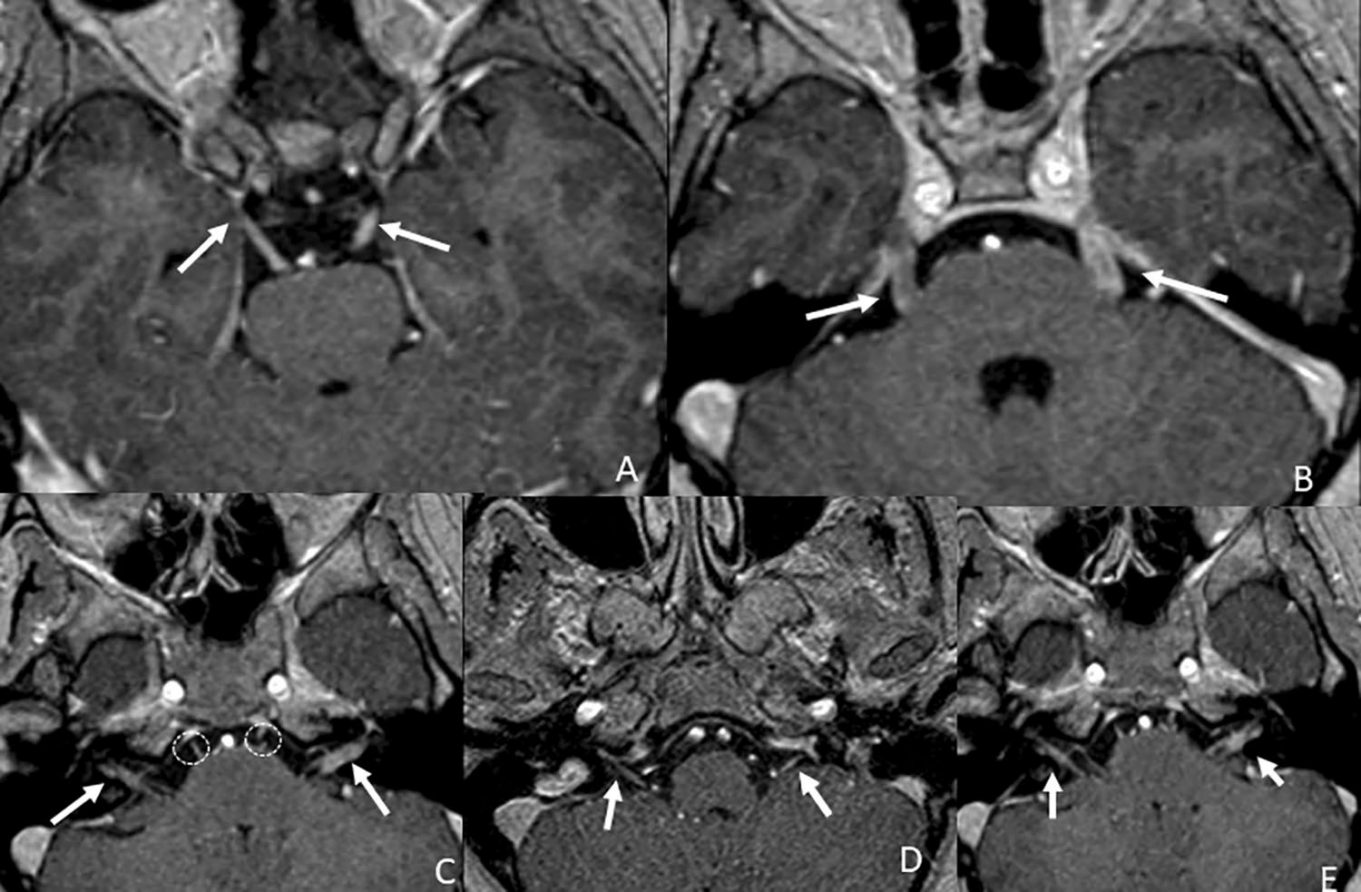
Axial T1 3D post-contrastographic weighted images show a thickened and asymmetrical but bilateral pathological enhancement of the III (**a**, arrows), V (**b**, arrows), VI (**c**, circles), VII–VIII (**c**, arrows), IX–X (**d**, arrows) and of the XII (**f**, arrows) pair of cranial nerves in a patient affected by Lyme disease

The mechanisms leading to CNE are different. In tuberculosis, the pathological nerve enhancement appears to be related to ischemia secondary to vasculitis or nerve entrapment by the exudates from the basal cisterns [15, 45], and therefore, it usually appears as bilateral and thickened, involving CN in their cisternal segments. In Lyme disease, cranial nerve involvement may be related to different processes, such as direct spirochetal invasion [46], vasculitis [16], or an immune-mediated condition [47] usually involving multiple cranial nerves with a bilateral thickening pattern (Fig. 10).

**Fig. 11:**
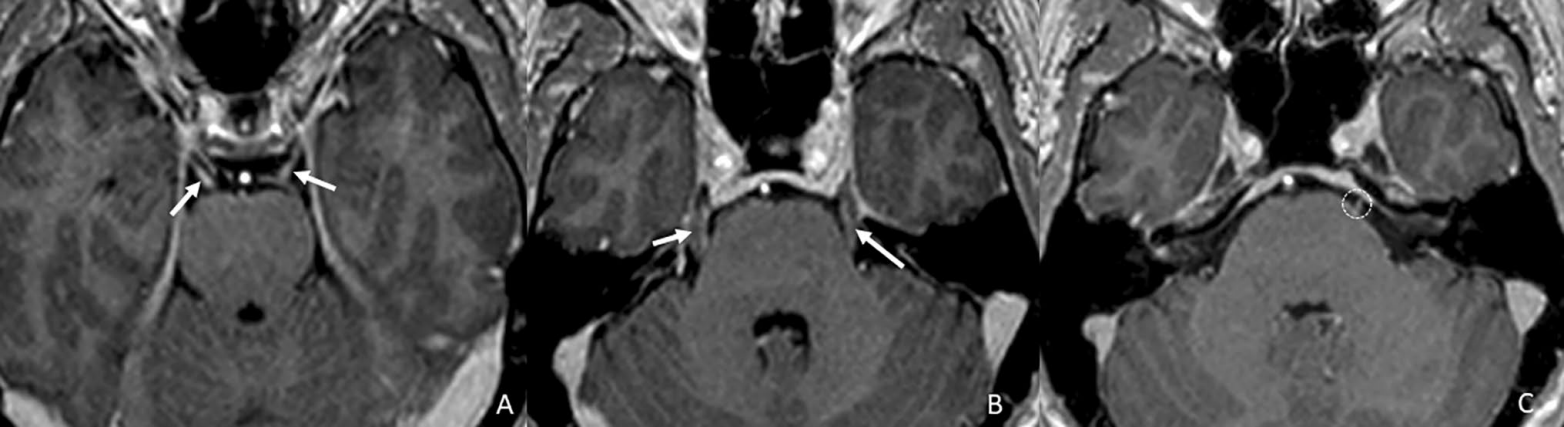
Axial T1 3D post-contrastographic weighted images showing bilateral linear enhancement of the III (**a**, arrows), and V (**b**, arrows) pair of cranial nerves and of the left VI CN (**c**, circle) and the right VII–VIII CN (**c**, arrow) in a patient with neurosyphilis

Also, in neurosyphilis infection, multiple cranial nerves are involved, and inflammation could represent the cause of the loss of BNB structural integrity [48–50] (Fig. 11). The central nervous system infection in Gradenigo's syndrome, also known as petrous apicitis and frequently caused by Pseudomonas and Enterococcus, is caused by contiguous infective dural invasion, which results in unilateral and thickened enhanced CN V and VI. The classic triad of symptoms is represented by suppurative otitis media, pain in the distribution territory of the trigeminal nerve, and abducens nerve palsy [51].

Finally, fungal infections, such as aspergillosis and mucor mycosis, are particularly prone to perineural involvement; in particular, mucor mycosis presents with a thin and unilateral pattern of cranial nerve enhancement; however, its pathogenesis is not well understood [52]*;* it could be possibly related to meningeal enhancement or to the presence of phlegmonous soft tissue along the cranial nerve [40]. Infection commonly begins in the paranasal sinuses and then spreads in the intracranial compartment along nerves and adjacent structures, being the CN II and the CNs, into the cavernous sinus [53]. If signal alterations extend posteriorly in the area of the maxillary sinuses, they may mimic the appearance of perineural spread along the V2 segment, and if the infra-temporal fossa is also involved, it could be mistaken for perineural spread along the V3 distribution.

### Inflammatory and immune-mediated related enhancement

Inflammatory and immune-mediated diseases can be related to different patterns of CNE due to different pathogenic mechanisms, which are most of the time still not clear. Usually, the pattern is thickened when inflammatory and granulomatous infiltrates are present, whereas it is linear if related to a demyelinating process in the acute phase. It is difficult to label them by location, as, with some exceptions, they can present with both a unilateral and bilateral pattern.

#### Demyelinating disorders

**Fig. 12:**
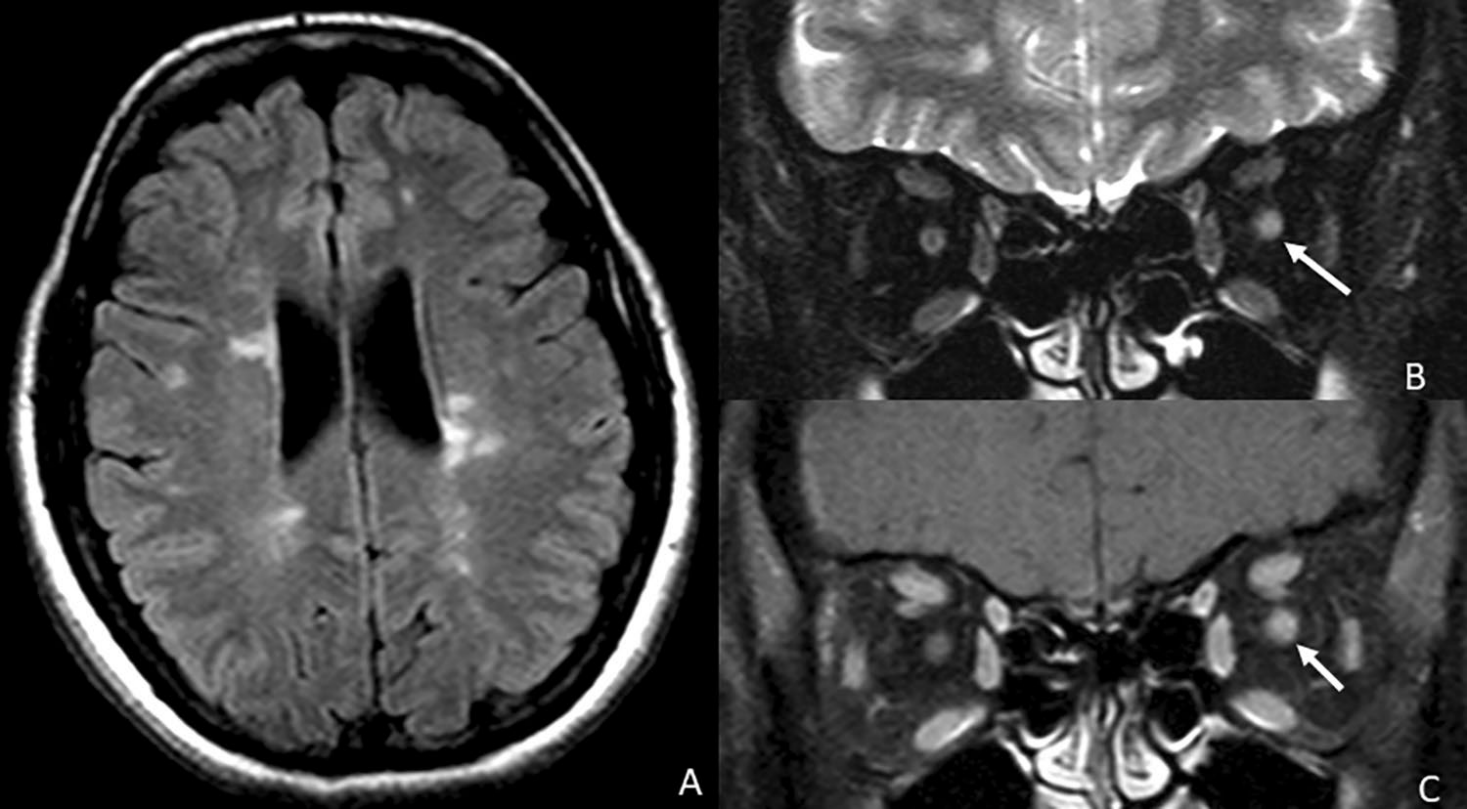
A case of a patient affected by multiple sclerosis with acute optic neuritis. **A** Axial FLAIR-weighted image showing multiple sclerosis-related hyperintense lesions in the periventricular white matter. **B** Coronal STIR-weighted image showing oedematous appearance of the left optic nerve in its intraorbital retrobulbar segment (arrow). That shows linear enhancement after contrast medium administration in **c** coronal T1 FS post-contrastographic weighted image (arrow)

**Fig. 13:**
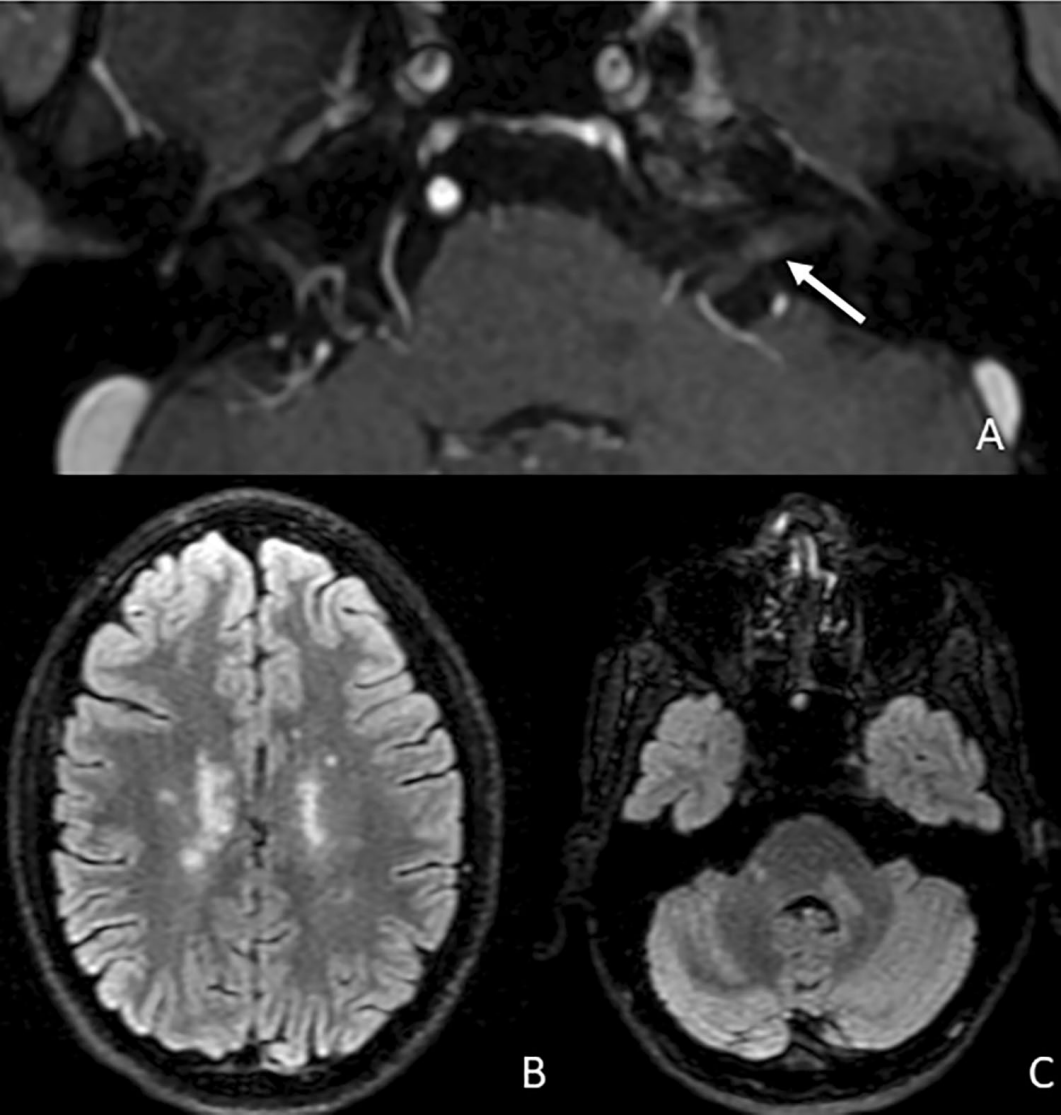
**A** Axial T1 3D post-contrast image showing a slight linear enhancement of the left VII–VIII cranial nerves in the internal acoustic canal (arrow), in a patient affected by multiple sclerosis with lesions in supratentorial (**b**. axial fat sat FLAIR) and subtentorial (**c**. axial Fat sat FLAIR) white matter

CNE is often related to an immune-mediated disorder. In this group, the cranial nerve most frequently involved is the II pair with the typical optic neuritis (Fig. 12). Radiologically, optic neuritis is characterized by an acute swelling and enhancement of the optic nerve, usually monolateral, in a short segment if related to multiple sclerosis (MS) or bilateral and longitudinally extensive if related to neuromyelitis optica (NMO) or anti-MOG encephalomyelitis. The involvement and consequently enhancement of the optic nerve in demyelinating spectrum disorders is easily explained by its diencephalic origin and oligodendrocyte myelination, which make it an extension of the central nervous system. In contrast, it is more difficult to explain the enhancement of other cranial nerves in MS that, even if rare, may occur (Fig. 13). It has been hypothesized that anterograde trans-synaptic neurodegeneration or inflammatory extension is a pathogenic mechanism, supported by the fact that, for example, the V cranial nerve enhancement is usually next to a lesion in the pontine entry zone of the trigeminal root [13, 54, 55].

#### Guillain–Barré syndrome

Guillain–Barré syndrome and its variant, the Miller-Fisher syndrome, can show cranial nerve involvement, which is clinically associated with ophthalmoplegia, ataxia, and areflexia [56]. The CNE, characterized by a prevalent bilateral thickened pattern, is the result of an immuno-inflammatory process caused by complement activation that leads to an anti-ganglioside antibody-mediated neuropathy [57].

#### Tolosa–Hunt syndrome

Tolosa–Hunt syndrome is a granulomatous inflammatory disorder of the cavernous sinus that also affects the orbit and superior orbital fissure. Retro-orbital pain and ophthalmoplegia, which may clinically resemble migrating ophthalmoplegia, define this syndrome.

**Fig. 14:**
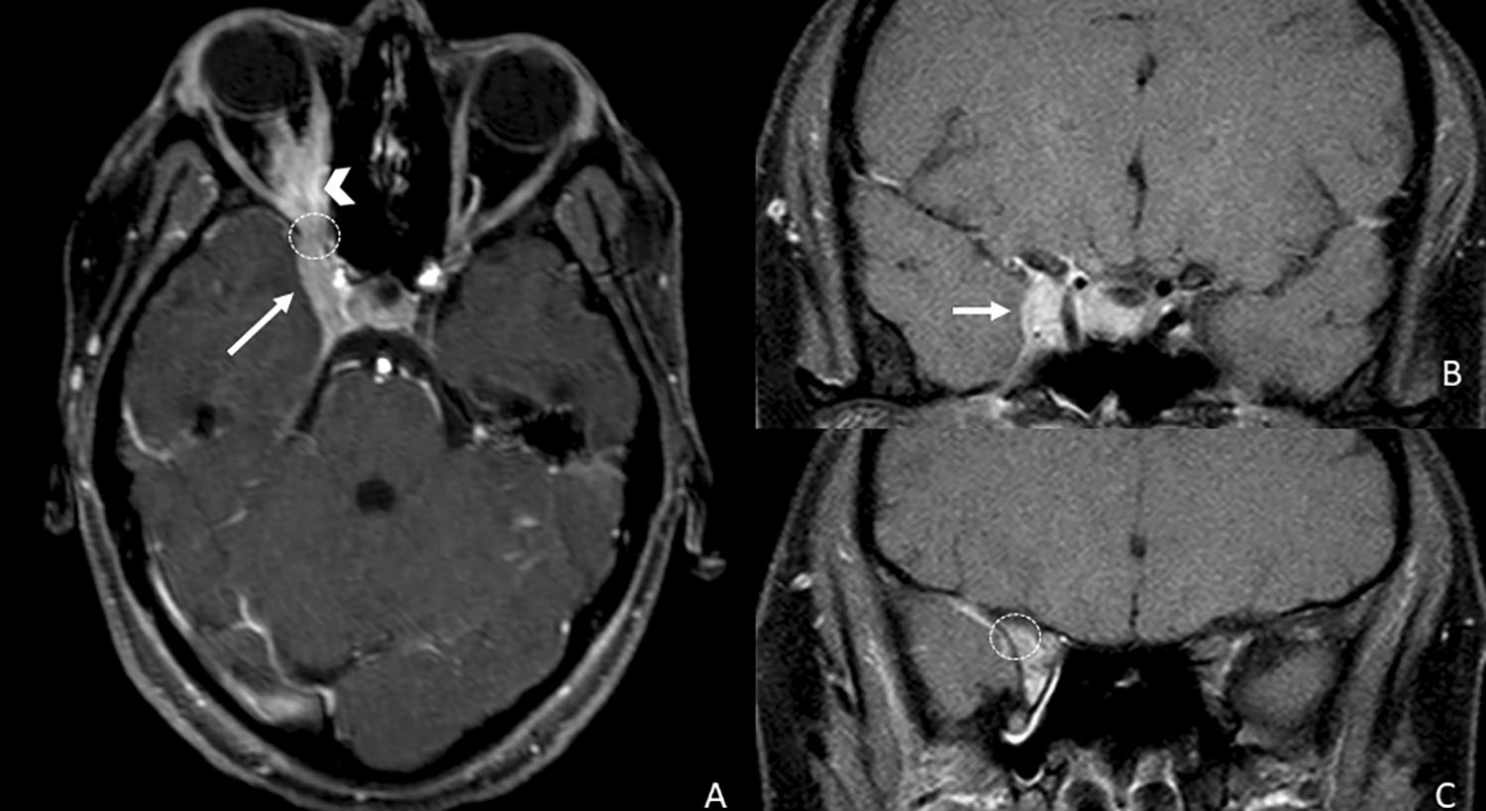
Axial (**a**) and coronal (**b**, **c**) T1 Fat Sat 3D post-contrastographic weighted images, showing a pathological enhancing tissue occupying the right cavernous sinus (**a**, **b** arrow), the right superior orbital fissure (**a**, **c** circle) and the right orbital apex (**a**, arrowhead) in a patient with Tolosa–Hunt Syndrome

The involvement of the cranial nerves is thus related to the presence of inflammatory tissue that invades the orbital apex with subsequent thickening and ipsilateral enhancement of the optic, oculomotor, and ophthalmic branches of the trigeminal nerve. On MRI, it is possible to observe an abnormal increase in soft tissue in the ipsilateral cavernous sinus [23, 58] (Fig. 14).

#### Ophthalmoplegic migraine

Ophthalmoplegic migraine is a rare syndrome characterized by head pain and ophthalmoplegia. Nowadays, it seems that it not a migraine variant but rather a form of cranial neuropathy that triggers headaches secondarily. The third cranial nerve is most affected by thickening of its cisternal portion. A proposed pathogenetic hypothesis is related to a demyelinating neuropathy; however, the exact pathologic mechanism is still unclear [22].

#### Sarcoidosis

**Fig. 15:**
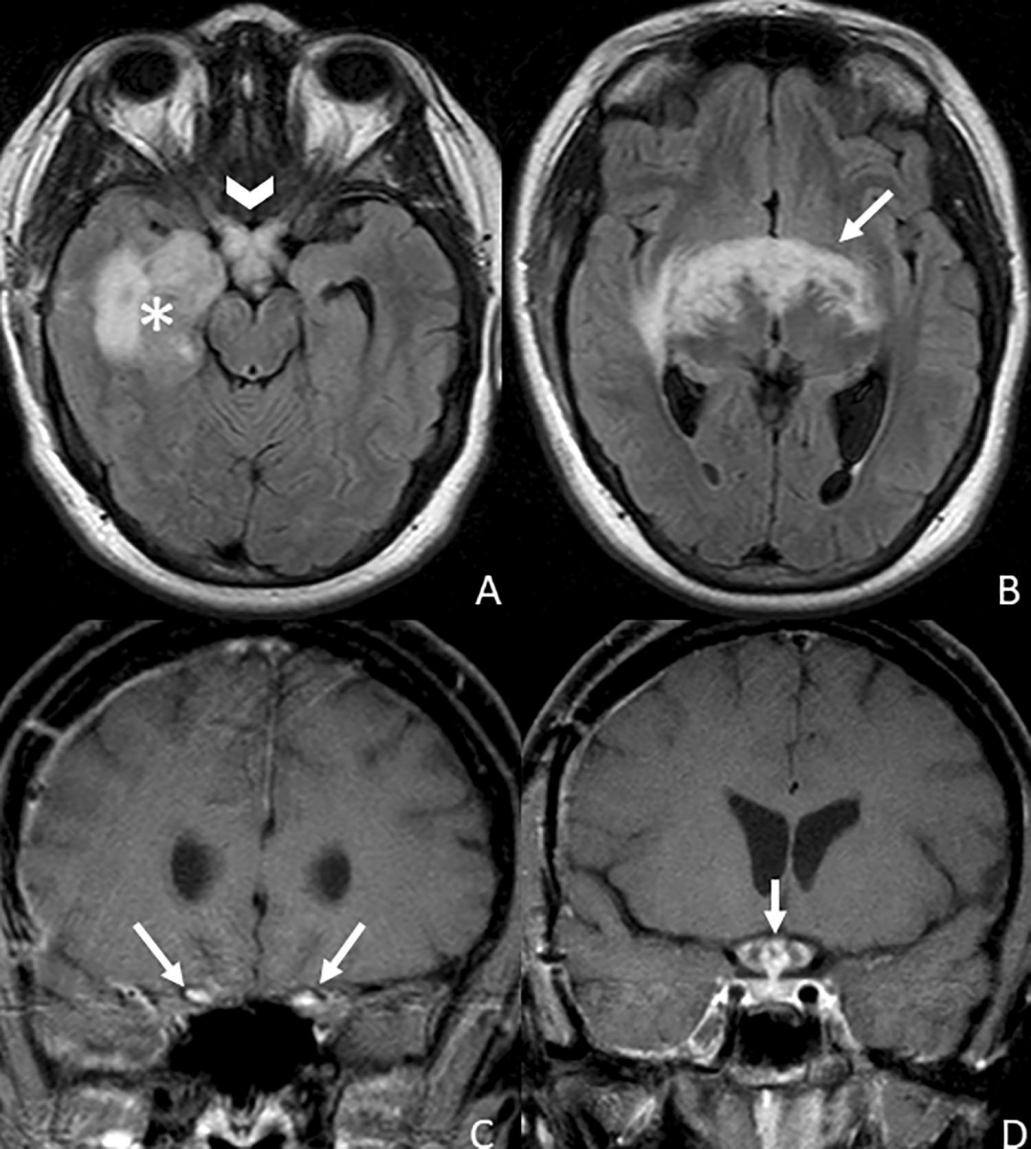
Axial FLAIR images (**a**, **b**) show marked hyperintensity of the region of the optic chiasm (arrowhead), involving the optic tracts (arrows) and extending into the right temporal lobe (asterisk) in a patient with neurosarcoidosis. This finding is correlated with a pathological bilateral thickened enhancement of the intracranial segment of the optic nerves (**c**, arrows) and of the optic chiasm (**d**, arrow)

Sarcoidosis is a chronic systemic disease of still unknown etiology. It is characterized by the presence of non-caseous granulomas, which may infiltrate different organs, including the CNS, with cranial nerves as the most involved site [59, 60]. All the cranial nerves can be involved; however, the most affected are the CN II and VII–VIII, often with unilateral enhancement, but in 30% of cases it can be bilateral [61, 62] (Fig. 15). The underlying pathogenesis of nerve enhancement and thickening is not clear; nevertheless, several theories have been proposed, including epineural or perineural granulomatous inflammation of the extra-cranial portion of the nerve or leptomeningeal granulomatous inflammation with consequent secondary compression and neural suffering [61, 63].

#### Wegner’s granulomatosis

Granulomatosis with polyangiitis, or Wegener's granulomatosis, is a systemic autoimmune disease characterized by the presence of non-caseous granulomatosis, which generally involves the kidneys and respiratory system [34, 64]. CNS involvement is infrequent, manifesting mainly with pachymeningitis and cranial nerve palsies, which appear thickened on imaging unilaterally or, more often, bilaterally [65]. There are several explanations that justify CNS involvement: primarily, it may be linked to the spread of the inflammatory process from the paranasal sinuses to the fronto-nasal meninges or through the orbit, with consequent involvement of the CN; other alternative theories include CNS vasculitis and/or the formation of distant granulomatous lesions in the CNS [65, 66].

#### Systemic lupus erythematosus and Sjogren’s syndrome

Systemic lupus erythematosus is a chronic, systemic autoimmune disease that rarely involves cranial nerves, manifesting mainly as unilateral third cranial nerve palsy, which shows linear enhancement [67, 68] and may be related to microvascular insults [67, 69, 70]. Sjogren's syndrome is a chronic, systemic, immune-mediated inflammatory disease as well [71]; when cranial nerve enhancement occurs, it may be unilateral or bilateral with multiple nerves affected and a thickened aspect in the acute phase [20]. Again, the pathogenetic mechanism is unclear, and a vasculitis cause or lymphocytic infiltrates-related changes have been proposed.

## Conclusions

We identified four patterns of CNE, provided flowcharts to navigate through the different disease and gave technical MRI notes to properly study CNE.

In conclusion, our approach to cranial nerve enhancement aimed to be an easy tool immediately applicable to clinical practice for converting challenging findings into specific pathological patterns.

## Data Availability

The datasets used and/or analyzed during the current study are available from the corresponding author on reasonable request.
